# The Role of Ethnicity and Migration in Perinatal Inequalities: A Retrospective Cohort Study

**DOI:** 10.1111/1471-0528.70169

**Published:** 2026-01-29

**Authors:** Hannah Rayment‐Jones, Sam Burton, Laura Bridle, Yahye Mohamud, Abigail Easter, Paul Seed, The eLIXIR Born in South London Partnership, Lucilla Poston, Laura A. Magee, Robert Stewart, David Edwards, Mark Ashworth, Ingrid Wolfe, Cheryl Gillett, Michael Absoud, Lucy Pickard, Amanda Grey, Sarah Spring, Toyin Kazeem, Amelia Jewell, Matthew Broadbent, Finola Higgins, Leonardo de Jongh, Tisha Dasgupta, Jane Sandall

**Affiliations:** ^1^ Department of Women and Children's Health King's College London London UK; ^2^ School of Psychology Liverpool John Moores University Liverpool UK; ^3^ Kings College Hospital NHS Trust London UK; ^4^ Faculty of Life Sciences and Medicine King's College London London UK

**Keywords:** ethnicity, inequality, language barrier, migration, nativity, perinatal

## Abstract

**Objective:**

To examine ethnic disparities in perinatal outcomes and the role of migration factors.

**Design:**

Retrospective cohort.

**Setting:**

Two maternity services in South London, UK.

**Population or Sample:**

Women birthing singleton infants between 24 and 43 weeks' gestation (2018–2023).

**Methods:**

Linked electronic health records were analysed using generalised linear mixed models (GLMMs) with Poisson distribution to estimate adjusted risk ratios (aRR) and 95% confidence intervals (CI) by ethnicity, migration, interpreter need, and country‐of‐origin income, adjusting for socioeconomic deprivation and medical risk.

**Main Outcome Measures:**

Emergency caesarean, haemorrhage, preterm birth, low birthweight, low Apgar score, stillbirth or neonatal death.

**Results:**

Among 44 634 births, compared with White women, emergency caesarean risk was higher for Asian (aRR 1.22, 95% CI 1.14–1.30, *p* < 0.001) and Black women (1.16, 1.10–1.23, *p* < 0.001). Haemorrhage was higher for Asian women (1.12, 1.02–1.23, *p* = 0.021), those needing interpretation (1.16, 1.06–1.27, *p* < 0.001), and lower for Mixed ethnicity women (0.86, 0.74–0.99, *p* = 0.038). Infants of Black women had elevated risks of preterm birth (1.23, 1.13–1.34, *p* < 0.001), low birthweight (1.74, 1.60–1.89, *p* < 0.001), low Apgar (2.06, 1.71–2.48, *p* < 0.001), and stillbirth/neonatal death (1.57, 1.21–2.05, *p* < 0.001). Asian infants had increased risks of preterm birth (1.19, 1.07–1.33, *p* = 0.002) and low birthweight (1.69, 1.52–1.87, *p* < 0.001). Foreign‐born women had lower risks of low birthweight (0.71, 0.62–0.81, *p* < 0.001) but higher risks of low Apgar (1.24, 1.06–1.46, *p* = 0.009) and stillbirth/neonatal death (1.33, 1.07–1.65, *p* = 0.011). Risks were highest for ethnic minority, foreign‐born women, though effect sizes were modest.

**Conclusions:**

Ethnic minority and foreign‐born women, particularly from LMICs or needing interpreters, face elevated risks with modest clinical impact.

## 1 Introduction

The quality and safety of maternity services have become central United Kingdom (UK) policy in recent years, following independent investigations into maternity and neonatal care at several NHS Trusts [1]. The National maternity inspection programme by the Care Quality Commission (CQC) has identified widespread issues, with 47% of maternity services in England currently requiring improvements in safety [2]. A decade ago, the National Maternity Safety Ambition was launched with the goal of halving the rates of stillbirths, neonatal and maternal deaths, and birth‐related brain injuries by 2025 [3]. Despite some progress, recent data show a rise in stillbirth and maternal mortality rates and persistent ethnic disparities across perinatal health outcomes [4, 5]. Adverse outcomes are notably higher among women and infants from Black and South Asian ethnic groups, as well as those experiencing social deprivation [4, 6, 7].

Current evidence suggests that these ethnic disparities cannot be attributed solely to socioeconomic deprivation; rather, they are influenced by intersecting factors such as unequal access to healthcare resources, geographic disparities, language barriers, individual and systemic racism [8, 9, 10]. There is conflicting evidence regarding whether recent migrant women experience better perinatal health compared to their native‐born ethnocultural counterparts or socioeconomically similar non‐immigrant White women [11, 12]. This phenomenon, often referred to as the “healthy migrant effect,” stems from the notion that those who choose or are able to emigrate tend to be younger, healthier, and better educated, as well as benefiting from healthier lifestyles and protective cultural and social factors [13, 14]. Additionally, they may have less exposure to structural and systemic discrimination [15]. The effect is thought to diminish over time in the host country and may not apply to forcibly displaced populations, such as refugees and asylum seekers [13, 16]. Nevertheless, evidence of the applicability of the ‘healthy migrant effect’ on maternal and infant health, particularly in the UK context, is sparse. It is not well understood how migration and acculturation influence ethnic inequalities, highlighting the need for more nuanced analyses to understand the underpinning mechanisms.

The NHS Race and Health Observatory's review on ethnic inequalities in healthcare [17] emphasised the need for advanced quantitative analyses to better comprehend the patterns of maternity outcomes and the intersection of inequalities across ethnicity, socioeconomic status, and other factors. This study aims to address evidence gaps by examining the extent of ethnic disparities in adverse perinatal outcomes within a multi‐ethnic, inner‐city population in South London. Using routinely collected, linked health records, we assess whether these disparities persist after adjusting for socioeconomic deprivation and clinical risk factors. We also explore how migration‐related variables: maternal migration, income level of country of birth, and recorded need for English language interpretation, may contribute to disparities between and within ethnic groups. This approach supports a more nuanced understanding of inequalities and will help inform future research priorities and targeted interventions to improve maternal and perinatal outcomes in the UK.

## 2 Methods

### 2.1 Research Aims

1. To compare the risk of adverse perinatal outcomes across ethnic groups, adjusting for socioeconomic deprivation and clinical risk factors.
2. To assess whether migration‐related factors—maternal migration, English proficiency (based on interpreter need), and income classification of country of birth—were independently associated with adverse maternal or infant outcomes.

### 2.2 Data Sources

We used linked, routinely collected data from the Early Life Cross‐Linkage in Research (eLIXIR‐BiSL) cohort partnership, comprising pseudonymised records from two acute and one mental health NHS Trust in South London [18, 19]. The dataset included 56 690 women and 67 308 pregnancies (October 2018–October 2023). Analyses were limited to 44 634 singleton pregnancies with complete booking and delivery records. Women were followed longitudinally from their maternity booking appointment through to delivery, with baseline exposures measured prior to outcome occurrence.

### 2.3 Measures

Ethnicity was categorised using the Office for National Statistics (ONS) broad categories [20]:

- White: Includes English, Welsh, Scottish, Northern Irish, British and other white
- Mixed: Includes mixed or multiple ethnic groups
- Asian or Asian British: Includes Asian, Asian British, and Asian Welsh
- Black or Black British: Includes Black, Black British, Black Welsh, Caribbean, or African
- Other: Includes any other ethnic group or where ethnicity was not documented

Socioeconomic status was measured using English Index of Multiple Deprivation quintiles, a method used to measure social and economic deprivation in small areas [21]. Education was excluded due to > 60% missing data to reduce bias from differential missingness.

Any pre‐existing medical risk (including but not limited to previous antepartum haemorrhage, preterm birth, stillbirth, placental abruption, diabetes), including obstetric risk‐factors identified prior to birth was determined by a healthcare professional at the initial maternity booking appointment and therefore specific conditions are not defined. Women with identified risk were allocated to obstetric‐led care; low‐risk women received midwife‐led care. Those developing complications during pregnancy (e.g., gestational diabetes, pre‐eclampsia) were typically transferred to obstetric‐led care, though these changes were not consistently captured in the dataset.

Migration was measured using the woman/mother's migration (born in UK, not born in UK). Other migration‐related variables included English language proficiency (requiring interpreter, not requiring interpreter) and classification of country‐of‐origin income level (HIC, LMIC defined by the 2022 World Bank Gross National Income classification [22]).

### 2.4 Maternity and Infant Outcome Variables

Primary maternal outcomes were emergency caesarean section and obstetric haemorrhage > 1000 mL (antepartum or postpartum). Primary infant outcomes were preterm birth (< 37 weeks), low birthweight (< 2500 g), Apgar score ≤ 7 at 5 min, stillbirth (≥ 24 weeks), and neonatal death (< 28 days). These were selected based on consistency with National Maternity and Perinatal Audit (NMPA) [23] and English Maternity Morbidity Outcome Indicator definitions [24]. A secondary analysis examined whether migration‐related factors were associated with any of the above outcomes. All were coded as binary (yes/no); definitions are provided in Table S1.

### 2.5 Statistical Analysis

Analyses were conducted using R. Only singleton pregnancy episodes were included; multiple births (e.g., twins) and duplicate records were excluded. Women were included if they had booking appointment data. Women with multiple singleton pregnancies across the study period were retained, with clustering accounted for through fitting a random intercept for each woman, using generalised linear mixed models (GLMMs). Significance was set at a *p*‐value of < 0.05.

Relative risks (RRs) and 95% confidence intervals (CIs) were estimated using GLMMs with a Poisson distribution, log link, and a random intercept for each woman's ID to account for repeated pregnancies. This approach allowed for direct estimation of relative risks rather than odds ratios. Models were adjusted for maternal age, parity, BMI > 30 kg/m^2^, smoking status, previous caesarean section, IMD quintile, and pre‐existing medical risk status. Migration‐related factors were examined individually and stratified to explore differential patterns. Due to limited counts in smaller subgroups, we did not test formal interactions; this is noted as a limitation and area for future research. Medical risk status was included to reflect baseline clinical complexity, though it may also capture structural disadvantage and lie on the causal pathway. These findings should be interpreted with caution.

## 3 Findings

### 3.1 Demographics

Table 1 presents baseline maternal characteristics at booking by ethnic group (White, Mixed, Asian, Black, and Other). White women (*n* = 23 838) were most likely to be UK‐born (57.7%) and to have English as their primary language (75.7%) and had the lowest rate of interpreter need (4.0%). In contrast, the majority of Asian (*n* = 4501) and Black (*n* = 8890) women were born outside the UK (71.6% and 68.4%, respectively). Black women had the highest levels of pre‐existing medical risk (56.5%) and obesity (34.5%). Women in the ‘Any Other’ ethnic group (*n* = 2916) had the lowest proportion with English as a first language (38.9%) and the highest rate of interpreter need (23.6%) and social risk (25.8%).

**TABLE 1:** Maternal baseline characteristics.

Demographic *n* (%)	White *n* = 23 838	Mixed *N* = 2326	Asian *N* = 4501	Black *N* = 8890	Any other *N* = 2916
< 20 years	234 (0.98%)	73 (3.14%)	15 (0.33%)	152 (1.71%)	38 (1.30%)
Missing	0	0	0	0	0
Primiparous	13 532 (56.77%)	1181 (50.77%)	2465 (54.77%)	3292 (37.03%)	1460 (50.07%)
Missing	0	0	0	0	0
Born in the UK	13 759 (57.72%)	1329 (57.14%)	1280 (28.44%)	2811 (31.62%)	435 (14.92%)
Missing	690 (2.89%)	54 (2.32%)	131 (2.91%)	283 (3.18%)	86 (2.95%)
*Country of origin*					
High income	20 462 (85.84%)	1628 (69.99%)	1746 (38.79%)	3179 (35.76%)	921 (31.58%)
Low/middle income	2686 (11.27%)	644 (27.69%)	2624 (58.30%)	5428 (61.06%)	1909 (65.47%)
Missing	690 (2.89%)	54 (2.32%)	131 (2.91%)	283 (3.18%)	86 (2.95%)
English 1st language	18 043 (75.69%)	1770 (76.10%)	2763 (61.39%)	6718 (75.57%)	1134 (38.89%)
Missing	482 (2.02%)	33 (1.42%)	83 (1.84%)	184 (2.07%)	57 (1.95%)
Need for interpreter	953 (4.00%)	176 (7.57%)	453 (10.06%)	556 (6.25%)	689 (23.63%)
Missing	1125 (4.72%)	90 (3.87%)	192 (4.27%)	378 (4.25%)	109 (3.74%)
*Social deprivation (IMD quintile)*					
1st (most deprived)	3224 (13.52%)	497 (21.37%)	763 (16.95%)	2682 (30.17%)	629 (21.57%)
2nd	8192 (34.37%)	956 (41.10%)	1533 (34.06%)	4041 (45.46%)	1242 (42.59%)
3rd	6389 (26.80%)	468 (20.12%)	1016 (22.57%)	1372 (15.43%)	629 (21.57%)
4th	3526 (14.79%)	231 (9.93%)	649 (14.42%)	421 (4.74%)	238 (8.16%)
5th (least deprived)	2240 (9.40%)	125 (5.37%)	453 (10.06%)	140 (1.57%)	114 (3.91%)
Missing	267 (1.12%)	49 (2.11%)	87 (1.93%)	234 (2.63%)	64 (2.19%)
Obstetric risk at booking	5581 (23.41%)	567 (24.38%)	1108 (24.62%)	2973 (33.44%)	722 (24.76%)
Medical risk at booking	11 370 (47.70%)	1213 (52.15%)	2070 (45.99%)	5025 (56.52%)	1300 (44.58%)
Pre‐existing mental health conditions	7099 (29.78%)	729 (31.34%)	734 (16.31%)	1595 (17.94%)	529 (18.14%)
Any social risk factor at booking	1994 (8.36%)	394 (16.94%)	552 (12.26%)	1495 (16.82%)	753 (25.82%)
*BMI>/30 kg/m* ^*2*^					
Yes	3084 (12.94%)	482 (20.72%)	561 (12.46%)	3069 (34.52%)	502 (17.22%)
Missing	0	0	0	0	0
*Smoker at booking*					
Yes	1091 (4.58%)	161 (6.92%)	40 (0.89%)	250 (2.81%)	64 (2.19%)
Missing	1090 (4.57%)	100 (4.30%)	183 (4.07%)	394 (4.43%)	145 (4.97%)

### 3.2 Maternal Outcomes by Ethnicity

Table 2 presents risk ratios (RR) and adjusted risk ratios (aRR) for emergency caesarean section and obstetric haemorrhage by ethnicity, with White women as the reference group.

**TABLE 2:** RR's and aRR's for maternal emergency caesarean section and obstetric haemorrhage.

Ethnicity	*N* (%)	RR (95% CI)	*p*	aRR (95% CI)^a^	*p*	aRR (95% CI)^b^	*p*
*Emergency caesarean (n = 10 234)*							
White	5229 (21.94%)	Ref		Ref		Ref	
Any other	729 (25.00%)	1.14 (1.06, 1.21)	0.001	1.13 (1.05, 1.21)	0.001	1.09 (1.01, 1.18)	0.032
Black	2534 (28.50%)	1.28 (1.23, 1.34)	< 0.001	1.28 (1.23, 1.34)	< 0.001	1.16 (1.10, 1.23)	< 0.001
Mixed/Multiple	542 (23.30%)	1.07 (0.99, 1.16)	0.120	1.07 (0.99, 1.16)	0.090	1.03 (0.94, 1.13)	0.504
Asian	1200 (26.66%)	1.22 (1.15, 1.29)	< 0.001	1.22 (1.15, 1.28)	< 0.001	1.22 (1.14, 1.30)	< 0.001
*Obstetric haemorrhage (n = 4767)*							
White	2571 (10.79%)	Ref		Ref		Ref	
Any other	324 (11.11%)	1.03 (0.91, 1.15)	0.658	1.04 (0.92, 1.16)	0.548	1.00 (0.89, 1.13)	0.982
Black	1117 (12.56%)	1.15 (1.07, 1.23)	< 0.001	1.17 (1.09, 1.26)	< 0.001	1.09 (1.00, 1.17)	0.050
Mixed	211 (9.07%)	0.85 (0.74, 0.97)	0.023	0.86 (0.74, 0.98)	0.033	0.86 (0.74, 0.99)	0.038
Asian	544 (12.09%)	1.12 (1.02, 1.23)	0.0131	1.13 (1.03, 1.24)	0.011	1.12 (1.02, 1.23)	0.021

*Note:* For ethnicity categories: ^a^Adjusted for socioeconomic deprivation, ^b^Adjusted for socioeconomic deprivation, high medical risk status, previous c‐section, BMI and smoker at booking.

#### 3.2.1 Emergency Caesarean Section

After adjustment, Asian women had the highest risk of emergency caesarean birth compared to White women (aRR 1.22, 95% CI 1.14–1.30; *p* < 0.001), followed by Black women (aRR 1.16, 95% CI 1.10–1.23; *p* < 0.001) and women of ‘Any Other’ ethnicity (aRR 1.09, 95% CI 1.01–1.18; *p* = 0.032). No difference was observed for women of Mixed/Multiple ethnicity (aRR 1.03, 95% CI 0.94–1.13; *p* = 0.504).

#### 3.2.2 Obstetric Haemorrhage

Asian women also had a higher risk of obstetric haemorrhage compared to White women (aRR 1.12, 95% CI 1.02–1.23; *p* = 0.021), as did Black women (aRR 1.09, 95% CI 1.00–1.17; *p* = 0.050). Mixed/Multiple ethnicity women had a lower risk (aRR 0.86, 95% CI 0.74–0.99; *p* = 0.038), while no difference was found for women of ‘Any Other’ ethnicity (aRR 1.00, 95% CI 0.89–1.13; *p* = 0.982).

### 3.3 Maternal Outcomes by Ethnicity, Migration, Country of Origin Income Classification and Interpretation Need

Table 3 shows adjusted risk ratios (aRR) for emergency caesarean and obstetric haemorrhage by maternal ethnicity, migration, country of origin income classification and interpreter need, with UK‐born women as the reference group.

**TABLE 3:** aRR for maternal adverse outcomes by ethnicity, migration country of origin income classification and interpretation need.

	Emergency caesarean aRR^a^	*p*	Obstetric haemorrhage aRR^a^	*p*
*Born in the UK (All ethnic groups)*				
Yes	Ref		Ref	
No	1.04 (1.00, 1.09)	0.046	1.09 (1.02, 1.16)	0.007
*Foreign‐born country of origin*				
UK‐born low SES	Ref		Ref	
HIC	0.95 (0.89, 1.02)	0.139	1.10 (1.04, 1.16)	< 0.001
LMIC	1.02 (0.96, 1.08)	0.464	1.05 (1.00, 1.10)	0.066
*Foreign‐born: Interpreter required*				
UK‐born white	Ref		Ref	
No	1.11 (1.06, 1.17)	< 0.001	1.08 (1.04, 1.12)	< 0.001
Yes	1.16 (1.06, 1.27)	< 0.001	0.97 (0.91, 1.04)	0.456
*Ethnicity and migration*				
White UK‐born	Ref		Ref	
White foreign‐born	1.04 (0.98, 1.10)	0.172	1.08 (1.03, 1.13)	< 0.001
Any other foreign‐born	1.11 (1.01, 1.22)	0.026	1.05 (0.97, 1.13)	0.234
Black foreign‐born	1.18 (1.10, 1.27)	< 0.001	1.03 (0.97, 1.09)	0.378
Mixed/Multiple foreign‐born	1.15 (1.01, 1.31)	0.038	1.05 (0.94, 1.17)	0.336
Asian foreign‐born	1.23 (1.14, 1.33)	< 0.001	1.12 (1.05, 1.19)	0.001

^a^ Adjusted for maternal socioeconomic deprivation, high medical risk status, BMI, previous C‐section, and smoker at booking.

#### 3.3.1 Migration

Women born outside the UK had higher risks of emergency caesarean (aRR 1.04, 95% CI 1.00–1.09; *p* = 0.046) and obstetric haemorrhage (aRR 1.09, 95% CI 1.02–1.16; *p* = 0.007) compared with UK‐born women.

#### 3.3.2 Country of Origin Income Classification

No significant differences were observed in emergency caesarean section by country of birth income classification. Among foreign‐born women, only those from high‐income countries (HICs) had an elevated risk of obstetric haemorrhage (aRR 1.10, 95% CI 1.04–1.12; *p* < 0.001).

#### 3.3.3 Interpreter Need

Women requiring an interpreter had a higher risk of emergency caesarean section (aRR 1.16, 95% CI 1.06–1.27; *p* < 0.001), but not obstetric haemorrhage (aRR 0.97, 95% CI 0.91–1.04; *p* = 0.456). Foreign‐born women who were not recorded as requiring an interpreter also had increased risks of obstetric haemorrhage (aRR 1.08, 95% CI 1.04–1.12; *p* < 0.001) and emergency caesarean section (aRR 1.11, 95% CI 1.06–1.17; *p* < 0.001).

#### 3.3.4 Stratified Analyses by Ethnicity and Migration

Compared with UK‐born White women, the risk of emergency caesarean section was higher among foreign‐born women in most ethnic groups, including ‘Other ethnicity’ (aRR 1.11, 95% CI 1.01–1.22; *p* = 0.026), Black (aRR 1.18, 95% CI 1.10–1.27; *p* < 0.001), Mixed/Multiple ethnicities (aRR 1.15, 95% CI 1.01–1.31; *p* = 0.038), and Asian (aRR 1.23, 95% CI 1.14–1.33; *p* < 0.001), but not among foreign‐born White women (aRR 1.04, 95% CI 0.98–1.10). For obstetric haemorrhage, increased risks were observed for foreign‐born White (aRR 1.08, 95% CI 1.03–1.13; *p* < 0.001) and Asian women (aRR 1.12, 95% CI 1.05–1.19; *p* = 0.001), with no significant migration‐related differences in Black, Mixed/Multiple, or Other ethnic groups.

### 3.4 Infant Outcomes by Ethnicity

Table 4 reports risk ratios (RR) and adjusted risk ratios (aRR) for preterm birth, low birthweight, low Apgar score, and stillbirth or neonatal death by ethnicity, using infants of White women as the reference group.

**TABLE 4:** RR and aRR for preterm birth, low birth weight, low Apgar score and neonatal death or stillbirth.

Ethnicity	*N* (%)	RR	*p*	aRR^a^	*p*	aRR^b^	*p*
*Preterm Birth (n = 4009)*							
White	1934 (8.11%)	Ref		Ref		Ref	
Any other	257 (8.81%)	1.08 (0.95, 1.23)	0.235	1.08 (0.94, 1.23)	0.258	1.08 (0.94, 1.24)	0.251
Black	956 (10.75%)	1.31 (1.21, 1.42)	< 0.001	1.30 (1.20, 1.41)	< 0.001	1.23 (1.13, 1.34)	< 0.001
Mixed/Multiple	197 (8.47%)	1.05 (0.91, 1.22)	0.481	1.05 (0.91, 1.21)	0.502	1.01 (0.87, 1.18)	0.865
Asian	427 (9.49%)	1.17 (1.06, 1.30)	0.003	1.17 (1.05, 1.29)	0.004	1.19 (1.07, 1.33)	0.002
*Low Birthweight (n = 4152)*							
White	1763 (7.40%)	Ref		Ref		Ref	
Any other	270 (9.26%)	1.25 (1.09, 1.41)	< 0.001	1.23 (1.08, 1.40)	0.001	1.28 (1.11, 1.46)	< 0.001
Black	1122 (12.62%)	1.69 (1.56, 1.82)	< 0.001	1.66 (1.54, 1.80)	< 0.001	1.74 (1.60, 1.89)	< 0.001
Mixed/Multiple	211 (9.07%)	1.24 (1.07, 1.42)	0.003	1.23 (1.06, 1.42)	0.004	1.24 (1.07, 1.44)	0.004
Asian	527 (11.71%)	1.59 (1.54, 1.74)	< 0.001	1.58 (1.43, 1.74)	< 0.001	1.69 (1.52, 1.87)	< 0.001
*Low Apgar score (n = 777)*							
White	316 (1.33%)	Ref		Ref		Ref	
Any other	45 (1.54%)	1.16 (0.84, 1.57)	0.337	1.12 (0.81, 1.53)	0.470	1.17 (0.83, 1.60)	0.351
Black	262 (2.95%)	2.23 (1.89, 2.62)	< 0.001	2.19 (1.85, 2.60)	< 0.001	2.06 (1.71, 2.48)	< 0.001
Mixed/Multiple	41 (1.76%)	1.35 (0.96, 1.85)	0.069	1.34 (0.95, 1.83)	0.081	1.38 (0.98, 1.90)	0.057
Asian	61 (1.36%)	1.02 (0.77, 1.34)	0.867	1.02 (0.77, 1.33)	0.882	1.01 (0.75, 1.34)	0.935
*Neonatal death or stillbirth (n = 502)*							
White	199 (0.83%)	Ref		Ref		Ref	
Any other	34 (1.17%)	1.39 (0.95, 1.97)	0.075	1.38 (0.94, 1.96)	0.084	1.31 (0.85, 1.93)	0.202
Black	123 (1.38%)	1.64 (1.31, 2.05)	< 0.001	1.63 (1.28, 2.06)	< 0.001	1.57 (1.21, 2.05)	< 0.001
Mixed/Multiple	23 (0.99%)	1.20 (0.76, 1.80)	0.416	1.19 (0.75, 1.80)	0.426	1.07 (0.65, 1.67)	0.768
Asian	45 (1.00%)	1.20 (0.86, 1.64)	0.266	1.18 (0.85, 1.62)	0.308	1.39 (0.96, 1.91)	0.060

^a^ Adjusted for maternal socioeconomic deprivation.

^b^ Adjusted for maternal socioeconomic deprivation, high medical risk status, BMI, and smoker at booking.

#### 3.4.1 Preterm Birth (< 37 Weeks)

Infants of Black women had the highest adjusted risk of preterm birth (aRR 1.23, 95% CI 1.13–1.34; *p* < 0.001), followed by infants of Asian women (aRR 1.19, 95% CI 1.07–1.33; *p* = 0.002). No differences were found for infants of Mixed/Multiple ethnicity (aRR 1.01, 95% CI 0.87–1.18; *p* = 0.865) or of ‘Any Other’ ethnicity (aRR 1.08, 95% CI 0.94–1.24; *p* = 0.251).

#### 3.4.2 Low Birthweight (< 2500 g)

Higher risks were observed among infants of Black (aRR 1.74, 95% CI 1.60–1.89; *p* < 0.001), Asian (aRR 1.69, 95% CI 1.52–1.87; *p* < 0.001), Mixed/Multiple (aRR 1.24, 95% CI 1.07–1.44; *p* = 0.004), and ‘Any Other’ ethnicity mothers (aRR 1.28, 95% CI 1.11–1.46; *p* < 0.001), compared to White infants.

#### 3.4.3 Low Apgar Score (≤ 7 at 5 Min)

Infants of Black women had an increased risk of low Apgar score (aRR 2.06, 95% CI 1.71–2.48; *p* < 0.001). No other groups showed significant differences after adjustment.

#### 3.4.4 Stillbirth or Neonatal Death

Infants of Black women also had a higher adjusted risk of stillbirth or neonatal death (aRR 1.57, 95% CI 1.21–2.05; *p* < 0.001). No differences were observed for other ethnic groups.

### 3.5 Infant Outcomes by Ethnicity, Migration, Country of Origin Income Classification and Interpretation Need

Table 5 presents adjusted risk ratios (aRR) for adverse infant outcomes: preterm birth, low birthweight, low Apgar score at birth, and stillbirth or neonatal death by maternal migration, country of origin income classification, interpreter need, and ethnicity–migration combinations, with UK‐born women as the reference group.

**TABLE 5:** aRR for infant adverse outcomes by maternal ethnicity, migration country of origin, income classification and interpretation need.

	Preterm birth aRR^a^	*p*	Low birthweight aRR^a^	*p*	Low Apgar score aRR^a^	*p*	Stillbirth or neonatal death aRR^a^	*p*
*Mother born in the UK (All ethnic groups)*								
Yes	Ref		Ref		Ref		Ref	
No	0.94 (0.87, 1.01)	0.095	1.02 (0.95, 1.10)	0.558	1.24 (1.06, 1.46)	009	1.33 (1.07, 1.65)	0.011
*Maternal country of origin income*								
UK‐born low SES	Ref		Ref		Ref		Ref	
HIC	0.71 (0.62, 0.81)	< 0.001	0.69 (0.60, 0.78)	< 0.001	0.89 (0.67, 1.19)	0.443	0.81 (0.54, 1.21)	0.910
LMIC	0.85 (0.76, 0.95)	0.003	1.02 (0.92, 1.13)	0.723	1.44 (1.15, 1.80)	0.001	1.18 (0.85, 1.63)	0.323
*Foreign‐born: Interpreter required*								
UK‐born white	Ref		Ref		Ref		Ref	
No	1.02 (0.93, 1.12)	0.675	1.29 (1.17, 1.42)	< 0.001	1.42 (1.16, 1.73)	< 0.001	1.54 (1.15, 2.05)	0.004
Yes	1.01 (0.85, 1.20)	0.872	1.23 (1.03, 1.45)	0.019	1.24 (0.88, 1.76)	0.220	1.53 (0.95, 2.48)	0.080
*Maternal ethnicity and migration to UK*								
White UK‐born	Ref							
White foreign‐born	0.99 (0.88, 1.11)	0.828	0.99 (0.879, 1.12)	0.881	1.04 (0.81, 1.34)	0.757	1.29 (0.92, 1.83)	0.144
Any other foreign‐born	0.97 (0.80, 1.16)	0.724	1.24 (1.03, 1.49)	0.022	1.30 (0.88, 1.90)	0.184	2.09 (1.28, 3.39)	0.003
Black foreign‐born	1.13 (0.99, 1.30)	0.070	1.73 (1.51, 1.97)	< 0.001	2.50 (1.94, 3.23)	< 0.001	2.22 (1.50, 3.30)	< 0.001
Mixed/Multiple foreign‐born	0.87 (0.64, 1.15)	0.350	1.34 (1.03, 1.73)	0.025	1.68 (1.02, 2.77)	0.042	2.32 (1.21, 4.44)	0.011
Asian foreign‐born	1.13 (0.96, 1.33)	0.136	1.76 (1.51, 2.03)	< 0.001	1.02 (0.70, 1.49)	0.905	1.81 (1.15, 2.85)	0.011

^a^ Adjusted for maternal socioeconomic deprivation, high medical risk status, BMI, previous C‐section, and smoker at booking.

#### 3.5.1 Migration

Infants of foreign‐born women showed no significant difference in risk of preterm birth compared with those of UK‐born women (aRR 0.94, 95% CI 0.87–1.01; *p* = 0.095). Risks of low birthweight were lower among infants of foreign‐born mothers (aRR 0.71, 95% CI 0.62–0.81; *p* < 0.001). In contrast, the risks of low Apgar score (aRR 1.24, 95% CI 1.06–1.46; *p* = 0.009) and stillbirth or neonatal death (aRR 1.33, 95% CI 1.07–1.65; *p* = 0.011) were higher in infants of foreign‐born women.

#### 3.5.2 Country of Origin Income Classification

Compared with UK‐born women of low socioeconomic status, infants of foreign‐born women had a lower risk of preterm birth, particularly among those from high‐income countries (HICs) (aRR 0.71, 95% CI 0.62–0.81; *p* < 0.001) and, to a lesser extent, from low‐ and middle‐income countries (LMICs) (aRR 0.85, 95% CI 0.76–0.95; *p* = 0.003). For low birthweight, infants of HIC‐born mothers had lower risk (aRR 0.69, 95% CI 0.60–0.78; *p* < 0.001), whereas LMIC‐born mothers' infants had no difference (aRR 1.02, 95% CI 0.92–1.13; *p* = 0.723). In contrast, low Apgar score was more common among LMIC‐born mothers' infants (aRR 1.44, 95% CI 1.15–1.80; *p* = 0.001). Stillbirth or neonatal death was higher in infants of both HIC‐born mothers (aRR 1.29, 95% CI 1.17–1.42; *p* < 0.001) and LMIC‐born mothers (aRR 1.23, 95% CI 1.03–1.45; *p* = 0.019).

#### 3.5.3 Interpreter Need

Interpreter requirement was not associated with preterm birth (aRR 1.01, 95% CI 0.85–1.20; *p* = 0.872) or low birthweight (aRR 1.02, 95% CI 0.92–1.13; *p* = 0.723). However, foreign‐born women recorded as not needing an interpreter had increased risks of low Apgar score (aRR 1.42, 95% CI 1.16–1.73; *p* < 0.001) and stillbirth/neonatal death (aRR 1.54, 95% CI 1.15–2.05; *p* = 0.004).

#### 3.5.4 Stratified Analyses by Ethnicity and Migration

Compared with UK‐born white women, risk patterns varied across ethnic groups. No migration‐related differences were observed in preterm birth across ethnic groups. However, the risk of low birthweight was higher among infants of foreign‐born women in several groups, including the “Any Other” ethnic category (aRR 1.24, 95% CI 1.03–1.49; *p* = 0.022), Black (aRR 1.73, 95% CI 1.51–1.97; *p* < 0.001), Mixed/Multiple (aRR 1.34, 95% CI 1.03–1.73; *p* = 0.025), and Asian women (aRR 1.76, 95% CI 1.51–2.03; *p* < 0.001). Low Apgar scores were also more common among infants of foreign‐born Black (aRR 2.50, 95% CI 1.94–3.23; *p* < 0.001), Mixed/Multiple (aRR 2.22, 95% CI 1.50–3.30; *p* < 0.001), and Asian women (aRR 1.81, 95% CI 1.15–2.85; *p* = 0.011). Similarly, stillbirth or neonatal death was more frequent among infants of foreign‐born Black (aRR 1.68, 95% CI 1.02–2.77; *p* = 0.042), Mixed/Multiple (aRR 2.32, 95% CI 1.21–4.44; *p* = 0.011), and Asian women (aRR 1.81, 95% CI 1.15–2.85; *p* = 0.011).

## 4 Discussion

### 4.1 Main Findings

This study demonstrates persistent ethnic and migration‐related disparities in maternal and infant outcomes within an ethnically diverse urban UK population, despite universal healthcare access. Consistent with national patterns, Asian and Black women were more likely to be born outside the UK and require interpreter support [6]. The ‘Any Other’ ethnic group had the lowest English proficiency and highest interpreter need, highlighting its heterogeneity. Differences in maternal mental health issues and social risk factors at the first maternity appointment were also observed among ethnic groups. However, this is highly dependent on women's willingness to disclose sensitive or stigmatised issues, which may be influenced by cultural factors [25].

After adjusting for clinical and socioeconomic factors, Asian, Black, and ‘Any Other’ ethnicity women had a higher risk of emergency caesarean section. Asian women also had a higher risk of obstetric haemorrhage, while for Black women this association was borderline significant. Mixed/Multiple ethnicity women had a slightly lower risk of haemorrhage.

Infants of Black women were at higher risk of preterm birth, low birthweight, low Apgar score, and stillbirth or neonatal death. Infants of Asian women were more likely to have preterm birth and low birthweight, low Apgar scores, and stillbirth or neonatal death. Infants of Mixed and ‘Any Other’ ethnicity women had increased risk of low birthweight, with Mixed infants also at elevated risk of low Apgar scores and stillbirth or neonatal death.

Women born outside the UK, particularly those from LMICs, had a higher risk of emergency caesarean, obstetric haemorrhage, low Apgar scores, and stillbirth or neonatal death, though lower risks of preterm birth and low birthweight. Interpreter need was associated with a higher risk of emergency caesarean section.

### 4.2 Strengths and Limitations

This study used a retrospective cohort design based on routinely collected clinical data. While this enabled large‐scale, real‐world analysis across a diverse population, it also introduced limitations, including variability in data completeness, inconsistency in definitions across settings, and missing information for key confounders such as education and detailed migration history. Including migration, interpreter need, and country income classification added nuance beyond standard ethnic categories, although broad groups, particularly ‘Any Other’, may obscure important heterogeneity. Several covariates were inconsistently recorded; for example, education data were missing for over 60% of the sample, especially among migrant women. Although we stratified results by migration, interpreter need, and country income classification, we did not formally test for interaction effects because some subgroup combinations had small numbers, resulting in limited precision and unstable estimates. In response to reviewer feedback, we undertook an additional analysis comparing UK‐born women from socioeconomically deprived backgrounds with foreign‐born women from high‐ and low‐/middle‐income countries. This suggested that risks varied by both migration status and socioeconomic context, underscoring the need for larger datasets and intersectionality‐informed methods such as MAIHDA [26] to disentangle these relationships.

Medical and social risks were based on clinician assessments at booking, which may underestimate risk among women with limited prior healthcare contact. We included medical risk to account for baseline complexity, though this may attenuate associations with social disadvantage. Antenatal diagnoses such as gestational diabetes, pre‐eclampsia, and fetal growth restriction were inconsistently coded and therefore excluded, limiting assessment of potential underdiagnosis across groups. Care escalation pathways were not reliably recorded, restricting evaluation of whether risk was recognised and managed equitably. While all women in the cohort had a recorded booking appointment before delivery, some may have engaged late or presented in labour, prompting an emergency booking. Gestational age at first appointment was available but not analysed in this study. Delayed care initiation may contribute to adverse outcomes, particularly among recent migrants or those facing barriers to access. A separate study using this dataset is currently exploring antenatal engagement and risk factors among migrant women in greater depth.

Interpreter need was recorded but may not reflect the quality or adequacy of provision, and although not associated with most outcomes after adjustment (apart from emergency caesarean section), unmet need remains a concern. Additional barriers such as insecure immigration status, language stigma, and mistrust of healthcare were not captured but are likely to contribute to observed disparities.

Finally, statistical significance does not necessarily imply clinical relevance, particularly in large datasets where very small differences may reach *p* < 0.05. Several associations observed here were modest in magnitude, and some statistically significant findings may partly reflect the large sample size rather than meaningful clinical or public health impact. In line with ASA guidance [27], our interpretation prioritises effect sizes, confidence intervals, and consistency with prior evidence rather than *p*‐values alone. Given the number of comparisons, type I error is possible; although we did not apply formal multiplicity corrections to avoid inflating type II error, this decision may increase the likelihood of false‐positive findings. Results with confidence intervals bordering 1.00 or *p*‐values near 0.05 should therefore be viewed as suggestive rather than definitive. The dataset also spans the COVID‐19 pandemic, which may have influenced both access to care and perinatal outcomes. In addition, some stratified and exploratory analyses were limited by data sparsity, resulting in reduced precision and potentially unstable subgroup estimates.

### 4.3 Interpretation

The results highlight associations rather than causal effects between ethnicity and migration‐related factors and perinatal health outcomes. Socioeconomic deprivation plays a significant part in the inequalities observed, but reflecting the wider literature [6, 9, 28], did not account for all disparities. Given the large sample size, many associations were statistically significant but modest in magnitude, and this should be considered when interpreting the findings. Higher risks among non‐White ethnic groups and individuals born outside the UK are consistent with structural factors influencing maternal and infant health disparities. Black, Asian, and ‘Any Other’ ethnicity women had a higher likelihood of emergency caesarean section, consistent with previous research identifying increased obstetric risks in these groups across the UK, Europe, and the US [4, 11, 29, 30, 31, 32]. Factors such as maternal height variations, access to services, mental health, language barriers, communication challenges, and interpreter availability may contribute to these disparities [6, 29, 30, 31, 32, 33, 34, 35]. The attenuation of the association between Black ethnicity and obstetric haemorrhage after adjustment suggests that medical risk profiles, including higher obesity prevalence, are likely additional contributors. Results were consistent with UKOSS data [36], which demonstrated increased risks of severe maternal morbidity among Black African, Black Caribbean, Bangladeshi, Pakistani, and other non‐White women.

Disparities in infant health outcomes were also observed. Black infants faced elevated risks of preterm birth, low birth weight, low Apgar scores, and stillbirth/neonatal death. Asian infants experienced higher rates of preterm birth and low birth weight, though not stillbirth or neonatal death. These trends align with research from other high‐income countries [5, 28, 37, 38, 39]. Preterm birth and low birth weight lead to significantly higher healthcare costs due to prolonged neonatal intensive care unit stays, specialised medical care, and an increased risk of long‐term health complications. These expenses place a substantial financial burden on both families and healthcare systems, with the highest costs per patient associated with the most premature infants [40]. This analysis includes both spontaneous and iatrogenic preterm births, as the available data do not allow for reliable differentiation between subtypes. While preterm birth is often considered an adverse outcome, some iatrogenic preterm births are medically indicated and can be lifesaving for the mother or baby. In contrast, women who do not receive appropriate escalation of care may miss timely induction. Supporting this, Behboudi‐Gandevani et al. [11] found that migrant women were less likely to undergo induction of labour and more likely to experience conditions such as pregnancy‐induced hypertension, preeclampsia, macrosomia, and large‐for‐gestational‐age infants, outcomes that might be identified and managed with adequate antenatal care. Migrant women are also consistently found to have lower rates of adequate antenatal care access [41, 42, 43, 44, 45, 46]. While the association between Black ethnicity and stillbirth/neonatal death remained significant after adjustment, it attenuated for Asian ethnicity, suggesting that some differences may relate to differential access or response to medical care, highlighting opportunities for targeted interventions.

Foreign‐born White, Black, and ‘Any Other’ women were more likely to experience any adverse outcome, reinforcing the need for early identification and tailored support.

These findings align with international research showing that migrants face a higher likelihood of emergency caesarean births [47, 48, 49, 50, 51], and low Apgar scores [47, 50, 51, 52]. Some studies have suggested protective effects related to maternal country of birth, such as lower neonatal mortality rates among infants of Pakistan‐born mothers compared to UK‐born Pakistani mothers [53]. However, similar advantages were not observed for Black infants in this study. The impact of migration on outcomes such as obstetric haemorrhage, preterm birth, birth weight, and stillbirth remains inconsistent in the broader literature, necessitating further UK‐based research. A recent review summarising the evidence base on perinatal health outcomes of women with asylum seeker or refugee status demonstrated complex medical and social issues and experiences of racism, prejudice and stereotyping within perinatal healthcare [54]. Additionally, access and engagement with maternity care was obstructed by structural, organisational, social, personal and cultural barriers [54].

Across all models, women from low‐ or middle‐income countries had higher risks of adverse outcomes, echoing international findings [55, 56, 57]. However, it is important not to assume that women who come from a low‐income country have a low income. A systematic review found that the prevalence of low birth weight among migrants varies based on host country characteristics and the composition of migrant populations. It concluded that the primary determinants of migrant health are the societal factors of host countries, including legal protections, institutions, and health systems [58]. A more recent review [59] on the effect of limited access to antenatal care among undocumented migrant women in Europe found undocumented migrants were more likely to experience adverse pregnancy outcomes than documented migrants and registered citizens. A study based in Bradford, UK, found notable differences in perinatal health behaviours between first‐ and second‐generation migrant women, suggesting the impact of these differences on perinatal outcomes as a priority for future research [60]. Assessing intergenerational differences or migrant status was not possible in this study due to inadequate reporting in health records. Future analysis should focus on migrant status, distinctions between Low‐ and middle‐income countries of origin, and intergenerational differences using larger datasets.

Aside from emergency caesarean section, interpreter need was not significantly associated with most outcomes in adjusted models, which contrasts with existing literature that identifies language barriers as a risk factor for poor engagement with maternity services and adverse perinatal outcomes [4, 34, 54, 61]. This may reflect under‐recording or misclassification of interpreter need in routine health records, highlighting the need for further exploration.

Recent reviews [39, 54, 62, 63, 64] examining public health, policy, and clinical interventions to improve perinatal outcomes for ethnic minority and migrant women and their infants in high‐income countries have identified several potentially effective interventions. These include early pregnancy screening, group antenatal care, mental health support, midwife continuity of care integrating social welfare services such as housing, immigration assistance, food banks, and access to free clothing and baby equipment within maternity care, as well as removal of financial barriers to accessing care. These combined interventions are likely to be important for achieving the UN's Sustainable Development Goal of Universal Health Coverage by 2030, ensuring financial risk protection and access to quality essential healthcare services for all, regardless of migration status. A multi‐interventional approach, particularly one that integrates midwifery‐led models with community‐based services, could improve accessibility and engagement with services [64].

## 5 Conclusions

Disparities in perinatal outcomes persist across ethnic and migrant groups in the UK, with higher risks observed among Black, Asian, ‘Any Other’ ethnic groups, and women born outside the UK. However, many associations were modest, and given the large sample size, some statistically significant findings may reflect small absolute differences or chance rather than clinically meaningful effects. Interpretation should therefore emphasise effect sizes, confidence intervals, and consistent patterns across outcomes rather than isolated *p*‐values.

Addressing these inequalities requires a multi‐level approach that combines clinical action with broader policy reforms aimed at the social and structural drivers of risk. Improved and more granular recording of ethnicity, migration, and language‐related variables is essential to support nuanced and responsible interpretation of disparities and to avoid over‐emphasis on marginal effects. Researchers and policymakers should remain mindful that statistical significance does not inherently imply clinical relevance, and decisions about intervention must be grounded in contextual, clinical, and population‐health considerations.

Tailored, culturally safe and rights‐based approaches, including continuity of care models and targeted support for migrant women, are likely to be key components of an equitable maternity system. These strategies, alongside investment in workforce capacity, service infrastructure, and social support systems, are needed to reduce persistent inequalities in maternal and infant outcomes.

## Author Contributions

S.B., H.R.‐J., J.S., A.E., L.B. and P.S. conceived the study. S.B. and H.R.‐J. developed the research question and methodology. S.B. and Y.M. did data analysis and H.R.‐J. wrote the first draft. All authors contributed to the interpretation of results and revisions of the manuscript. All authors had full access to all the data in the study and had final responsibility for the decision to submit for publication. All authors have seen and approved of the final manuscript.

## Funding

This project is funded by the National Institute for Health and Care Research (NIHR) Applied Research Collaboration South London (NIHR ARC South London) at King's College Hospital NHS Foundation Trust. It was supported by the Early Life Cross Linkage in Research, Born in South London (eLIXIR‐BiSL) Partnership developed by an MRC Partnership Grant [MR/P003060/1] and the MRC Longitudinal Population Study Grant [MR/X009742/1]. The eLIXIR platform is also part‐supported by the National Institute for Health Research (NIHR) Biomedical Research Centre at the South London and Maudsley NHS Foundation Trust and King's College London. Dr. Hannah Rayment‐Jones is funded by a NIHR Advanced Fellowship (NIHR 303183). Yayhe Mohamud was supported by a Kings Undergraduate Research Fellowship (KURF). The views expressed in this article are those of the author(s) and not necessarily those of the NIHR, or the Department of Health and Social Care. The funders had no role in data collection, analysis, interpretation, report writing, or the decision to submit this report for publication. NIHR 301189.

## Ethics Statement

The Early Life Cross Linkage in Research, Born in South London (eLIXIR‐BiSL) Partnership has received ethical approval from the Oxfordshire Research Ethics Committee C (23/SC/0116) as an anonymised dataset for medical research and Section 251 support from the Confidential Advisory Group (18/CAG/0040).

## Conflicts of Interest

The authors declare no conflicts of interest.

## Supporting information


**Table S1:** Outcome measure and corresponding database variables.

## Data Availability

The data accessed by eLIXIR remain within an NHS firewall and governance is provided by the eLIXIR Oversight Committee reporting to relevant information governance clinical leads. Subject to these conditions, data access is encouraged and those interested should contact the eLIXIR Chief Investigator (Professor Lucilla Poston; lucilla.poston@kcl.ac.uk).
