# Supplementary material for: The Role of Ethnicity and Migration in Perinatal Inequalities: A Retrospective Cohort Study
Source: BJOG. 2026 Jan 29;133(6):1249–61. doi: 10.1111/1471-0528.70169 (PMC13040417; doi:10.1111/1471-0528.70169)
Supplement: Supplementary file 1 — Table S1: Outcome measure and corresponding database variables. [file BJO-133-1249-s001.docx]

| **Supplementary Table S1: Outcome measure and corresponding database variables** | | |
| --- | --- | --- |
| **Exposure/Outcome** | **Data set** | **How identified in database?** |
| Ethnicity  White  Any other  Black  Mixed/multiple  Asian | DS1 Booking dataset | MotherEthnicity categorised defined by ONS broad categories  Individuals who did not fall into the categories defined by ONS broad definitions, were categorised as “Any other” if they were categorised in the NHS data set as “Any Other ethnic group” |
| Born in UK | DS1 Booking dataset | CountryofBirth=”United Kingdom of Great Britain and Northern Ireland” |
| Foreign-born country of origin income level | DS1 Booking dataset | High income country or low and middle income country based off of CountryofBirth |
| Interpreter required | DS1 Booking dataset | InterpreterRequired=”Yes” |
| Obstetric risk at booking | DS1 Booking dataset | Free text search of ObstetricRiskFactor for any obstetric risk factors identified at booking appointment (e.g,. prior caesarean section). |
| Medical risk at booking | DS1 Booking dataset | Free text search of MedicalRiskFactor for any medical risk factors identified at booking appointment (e.g. neurological condition). |
| Pre-existing mental health conditions | DS1 Booking dataset | Free text search of MentalHealthRiskFactor for any mental health risk factors identified at booking appointment (e.g. severe mental illness). |
| Social risk factor at booking | DS1 Booking dataset | Free text search of SocialRiskFactor for any social risk factors identified at booking appointment (e.g. teen pregnancy, unborn child protection order). |
| Emergency caesarean section | DS2 Intrapartum and postnatal care | ModeOfDelivery = “Emergency an unspecified caesarean section” |
| Obstetric haemorrhage | DS2 Intrapartum and postnatal care | TotalBloodLoss >499ml |
| Pre-term birth | DS2 Intrapartum and postnatal care | GestationAtDeliveryWeeks <37 |
| Low Birth Weight | DS2 Intrapartum and postnatal care | BirthWeightGrams<2500g |
| Low Apgar Score | DS2 Intrapartum and postnatal care | APGARscore5minutes<7 at 5 minutes after birth |
| Neonatal death of stillbirth | DS2 Intrapartum and postnatal care | FinalBirthOutcome = “Livebirth” |
